# Room-temperature electrochemical water–gas shift reaction for high purity hydrogen production

**DOI:** 10.1038/s41467-018-07937-w

**Published:** 2019-01-08

**Authors:** Xiaoju Cui, Hai-Yan Su, Ruixue Chen, Liang Yu, Jinchao Dong, Chao Ma, Suheng Wang, Jianfeng Li, Fan Yang, Jianping Xiao, Mengtao Zhang, Ding Ma, Dehui Deng, Dong H. Zhang, Zhongqun Tian, Xinhe Bao

**Affiliations:** 1 State Key Laboratory of Catalysis, Collaborative Innovation Center of Chemistry for Energy Materials, Dalian Institute of Chemical Physics, Chinese Academy of Sciences, Dalian, 116023 China; 2 Collaborative Innovation Center of Chemistry for Energy Materials, College of Chemistry and Chemical Engineering, Xiamen University, Xiamen, 361005 China; 3 State Key Laboratory of Molecular Reaction Dynamics, Dalian Institute of Chemical Physics, Chinese Academy of Sciences, Dalian, 116023 China; 4 Center for High Resolution Electron Microscopy, College of Materials Science and Engineering, Hunan University, Changsha, 410082 China; 5 College of Chemistry and Molecular Engineering, Peking University, Beijing, 100871 China

## Abstract

Traditional water–gas shift reaction provides one primary route for industrial production of clean-energy hydrogen. However, this process operates at high temperatures and pressures, and requires additional separation of H_2_ from products containing CO_2_, CH_4_ and residual CO. Herein, we report a room-temperature electrochemical water–gas shift process for direct production of high purity hydrogen (over 99.99%) with a faradaic efficiency of approximately 100%. Through rational design of anode structure to facilitate CO diffusion and PtCu catalyst to optimize CO adsorption, the anodic onset potential is lowered to almost 0 volts versus the reversible hydrogen electrode at room temperature and atmospheric pressure. The optimized PtCu catalyst achieves a current density of 70.0 mA cm^−2^ at 0.6 volts which is over 12 times that of commercial Pt/C (40 wt.%) catalyst, and remains stable for even more than 475 h. This study opens a new and promising route of producing high purity hydrogen.

## Abstract (web summary)

Traditional water–gas shift reaction process is hindered by harsh reaction conditions and extra steps for hydrogen separation and purification. Here, the authors report a room temperature electrochemical water–gas shift process for direct production of high purity hydrogen with a faradaic efficiency of approximately 100%.

## Introduction

**Fig. 1:**
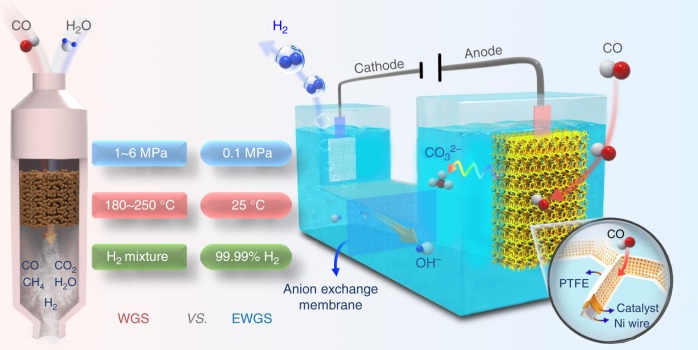
Schematic diagram of the EWGS process compared with the traditional WGS process. The cations K^+^ serve as the counter ions to balance the reaction

The water–gas shift (WGS) reaction, i.e., CO + H_2_O → H_2_ + CO_2_, is a key step in carbon-based energy processes for large-scale hydrogen production^1–5^. The process is typically operated at 1.0–6.0 MPa under high temperatures to overcome the sluggish reaction kinetics, though the reaction is mildly exothermic (∼41 kJ mol^−^^1^)^6,7^, and a lower temperature will favor the equilibrium moving toward hydrogen production^8^. As a compromise, industrial plants often use a two-step WGS reactor where the feed gas is firstly led through a high-temperature (320–450 °C) reactor to initiate the reaction with high rate and then a lower temperature (180–250 °C) reactor to further improve CO conversion^9–12^. Apart from the harsh conditions, H_2_ produced by the WGS reaction contains CO residuals (around 1–10%) and significant quantities of CO_2_ and CH_4_, etc., which needs extra processes of separation and purification (Fig. 1) so as not to affect the downstream applications^13^. As an example, a trace amount of CO (around 100 ppm) in H_2_ will seriously poison the Pt-based catalyst on the anode of proton exchange membrane fuel cell and reduce the performance significantly^14^. Therefore, the direct production of high purity hydrogen under mild conditions is more economical and eco-friendly but a challenging task, which requires innovative catalytic processes.

Electrochemically decoupling the WGS redox reaction to separated cathodic reduction reaction and anodic oxidation reaction in an electrolytic cell and using electric-potential as the driving force of the process is a promising approach of circumventing the aforementioned harsh conditions^15,16^. The catalyst and electrode structure significantly affect the intrinsic activity and micro-kinetics for the reaction, and thus need elaborate design towards higher reaction performance under milder conditions.

Herein, we report a room-temperature electrochemical water–gas shift (EWGS) process, where the water is reduced to H_2_ at the cathode and the CO is oxidized at the anode. Through rationally designing the anode structure and catalyst to promote the CO oxidation reaction, the anodic onset potential can be lowered to almost 0 V versus the reversible hydrogen electrode (RHE) at room temperature (25 °C) and atmospheric pressure, and high purity H_2_ (over 99.99%) is directly generated without the need of separation.

## Results

### Elaboration of the EWGS process

In the EWGS process as illustrated in Fig. 1, CO is oxidized on the anode through Eq. (1) (the cations K^+^ are omitted for clarity), where the product CO_2_ further reacts with hydroxide ion forming CO_3_^2^^−^ and thereby is transformed into a potassium carbonate (K_2_CO_3_) avoiding the pollution caused by CO_2_ emission. Meanwhile, H_2_ is produced from H_2_O reduction on the cathode through Eq. (2):

1 $${\mathrm{Anode:CO}} + {\mathrm{4OH}}^{\mathrm{ - }} \to {\mathrm{CO}}_{\mathrm{3}}^{{\mathrm{2 - }}} + {\mathrm{2H}}_{\mathrm{2}}{\mathrm{O}} + {\mathrm{2e}}^{\mathrm{ - }}{.}$$

2 $${\mathrm{Cathode:2H}}_{\mathrm{2}}{\mathrm{O}} + {\mathrm{2e}}^{\mathrm{ - }} \to {\mathrm{H}}_{\mathrm{2}} + {\mathrm{2OH}}^{\mathrm{ - }}{.}$$

3 $${\mathrm{Total:CO}} + {\mathrm{2OH}}^{\mathrm{ - }} \to {\mathrm{H}}_{\mathrm{2}} + {\mathrm{CO}}_{\mathrm{3}}^{{\mathrm{2}} - }{.}$$

Anion exchange membrane was employed for separating the cathode and anode, maintaining the balance of ion concentration of electrolyte, and hindering cross-contamination of the anodic (CO_2_) and cathodic (H_2_) reaction products in the system. Hence, the additional separation process as required in the traditional WGS reaction is precluded. In addition to H_2_ production, the other product K_2_CO_3_ in this EWGS is widely applied in the production of glass, soap, etc. in industry. The K_2_CO_3_ possesses higher added value than KOH because the production of K_2_CO_3_ in industry is from the reaction of KOH with CO_2_. Therefore, compared with the traditional WGS, the EWGS features a completely different catalytic reaction process and can operate at room temperature and ambient pressure, which opens a new avenue toward the direct production of high purity hydrogen with low-energy consumption.

### Optimization of the anode structure

When operating the EWGS reaction in aqueous electrolyte, we identified two key factors that significantly affect the performance of CO oxidation. One is the diffusion of CO in the water phase due to its low solubility. Increasing the local concentration of CO near the anode catalyst surface plays a crucial role in enhancing the reaction rate of CO oxidation. The other is the optimization of the anode catalyst to exhibit high activity and durability, that could efficiently decrease the overpotential for the EWGS process. Hence, we will address the two key factors as follows by optimizing the structure of the anode and the catalyst to enhance the energy conversion efficiency of the EWGS process.

**Fig. 2:**
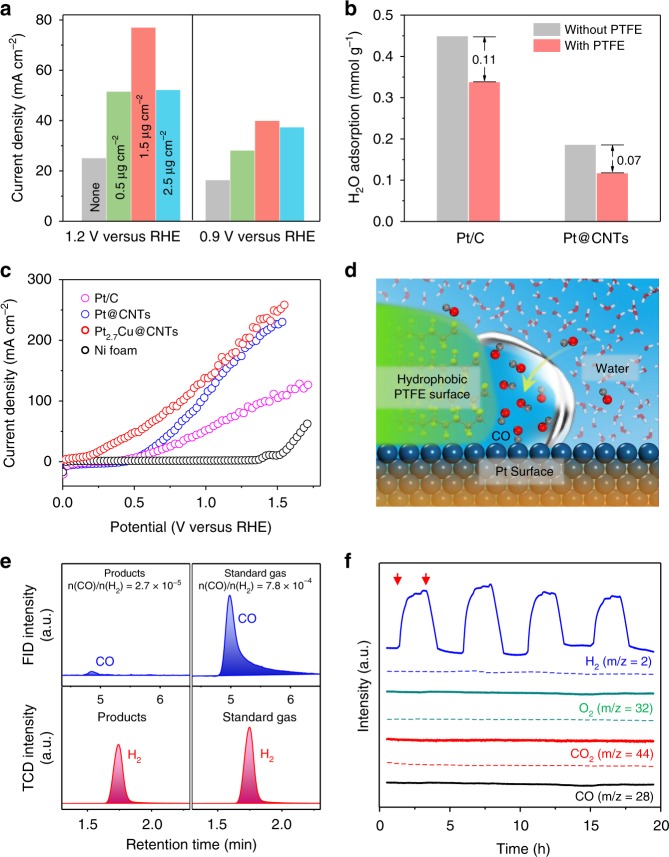
Optimization of anode structure and performance of the EWGS process. **a** Current densities of CO oxidation on Pt/C catalyst decorated with different content of PTFE at 0.9 and 1.2 V versus RHE, respectively. **b** Adsorption of H_2_O at 25 °C on Pt/C and Pt@CNTs with and without PTFE treatment detected by intelligent gravimetric analyzer. **c** Linear sweep voltammetry polarization curves of the CO oxidation catalyzed by Pt/C, Pt@CNTs, Pt_2.7_Cu@CNTs, and Ni foam, all decorated with 1.5 μg cm^−2^ PTFE. **d** Schematic illustration of solid/liquid/gas interfaces on the PTFE-decorated Pt surface. **e** Gas chromatography analysis of hydrogen purity for the cathodic products at a constant potential of 1.0 V versus RHE using Pt@CNTs as the anode catalyst, calibrated with the specific concentration of CO/H_2_ standard gas. **f** Mass spectrometry detection of the anodic (dotted line) and cathodic (solid line) products using Pt@CNTs as the anode catalyst at a constant current density of 20.0 mA cm^−2^. The red arrows denote starting and ending of CO inlet. All tests were carried out in CO-saturated 1 M KOH solution at 25 °C

The diffusion of CO to the active sites which depends on two properties of the anode: (i) pore structure and surface area of the anode and (ii) the accessibility of surface sites to CO. For the first point, we adopted a conductive Ni foam with rich porous structure and high surface area as the anode support for loading catalysts to ensure sufficient exposure of catalyst to reactants (Fig. 1 and Supplementary Fig. 1). For the second point, we employed the traditional carbon supported platinum WGS catalyst^17,18^ to optimize the anode structure. We deposited a hydrophobic polytetrafluoroethylene (PTFE) layer on commercial Pt/C (40 wt%) catalyst, aiming to build water-free compartments at the interface of PTFE and Pt to promote CO diffusion and facilitate CO colliding with the surface sites. Figure 2a and Supplementary Fig. 2a show that decoration of the catalyst with PTFE notably improves the CO oxidation activity and the anodic current density reaches the maximum at PTFE loading amount of 1.5 μg cm^−2^. Drop of current density at higher PTFE loading (2.5 μg cm^−2^) could be caused by the blocking of the active sites by the overloaded PTFE. Inspired by the feature of the hydrophobic PTFE layer, we supported Pt nanoparticles on well-graphitized carbon nanotubes (Pt@CNTs, 40 wt%), which are pretreated in hydrogen at 400 °C to remove the oxygen-containing groups from the CNTs surface and improve hydrophobicity. The adsorption amount of H_2_O on the Pt@CNTs is much lower than that on the pristine Pt/C and is further reduced upon PTFE treatment, as shown in the intelligent gravimetric analyzer tests (Fig. 2b). Linear sweep voltammetry polarization curves show that the current density of the Pt@CNTs anode catalyst reaches about twice that of the commercial Pt/C at potentials over 0.75 V (Fig. 2c and Supplementary Fig. 2b), though the size of the Pt nanoparticles supported on the CNTs is slightly larger than that of the commercial Pt/C (Supplementary Fig. 3). These results indicate that the presence of PTFE and CNTs significantly improves the hydrophobicity of the catalyst, which facilitates the diffusion and adsorption of CO and improves the anodic reaction activity via creating solid/liquid/gas interfaces (Fig. 2d).

During the activity measurements of the Pt@CNTs anode catalyst, the faradic efficiency for H_2_ production at the cathode maintains around 100% in a wide range of working potential and the H_2_ purity can reach over 99.99% (Fig. 2e and Supplementary Fig. 4). Only trace amounts (27 ppm) of CO are detected in the cathode output, which may be due to the microleakage from the anode side through the anion exchange membrane (Fig. 2e). In a cyclic process of open/close-circuit operation of the EWGS system, neither CO_2_ and O_2_ gases in the anode chamber nor CO gas in the cathode chamber is detected by mass spectrometry (Fig. 2f). These results confirm the direct production of high-purity H_2_ without additional separation through the EWGS process.

### Elucidation of the anodic reaction mechanism

**Fig. 3:**
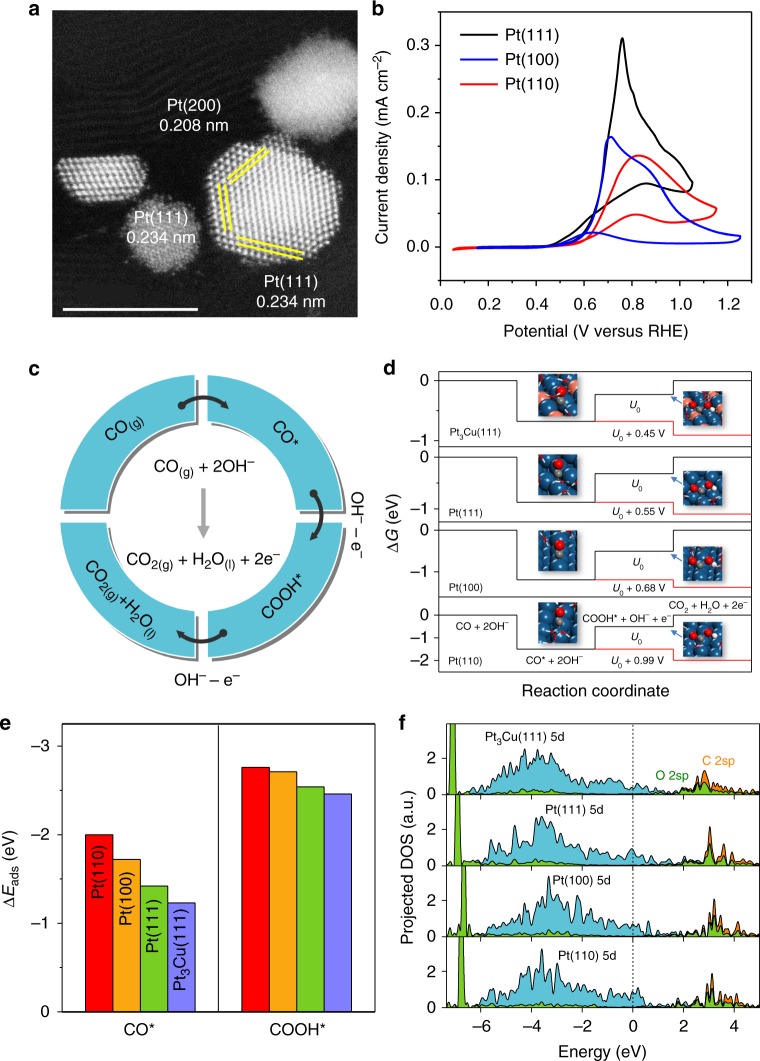
Insights into the reaction mechanism of different Pt facets towards the EWGS. **a** HAADF-STEM image of Pt@CNTs, scale bar: 5 nm. **b** CV test of Pt(111), Pt(110), and Pt(100) electrodes in CO-saturated 0.01 M KOH at 25 °C with a sweep rate of 50 mV s^−1^. **c** Reaction path of the EWGS process in alkaline solution. **d** Free energy diagrams of CO oxidation on Pt(110), Pt(100), Pt(111), and Pt_3_Cu(111) at the reversible potential (*U*_0_) of −0.16 V, and at the overpotentials that all reaction steps are downhill in free energy. **e**, **f** Comparison of the adsorption energies (Δ*E*_ads_) of CO and COOH and projected density of states of CO on Pt(110), Pt(100), Pt(111), and Pt_3_Cu(111) in water environment

Optimizing the activity of catalyst requires understanding of the structure and its relation to the catalytic reaction mechanism. Supported metal nanoparticles typically expose multiple crystallographic planes, which exhibit distinct catalytic activities. High-angle annular dark field scanning transmission electron microscopy (HAADF-STEM) images of the Pt@CNTs sample (Fig. 3a and Supplementary Figs. 5a, b) show that the Pt nanoparticles mainly expose the face-centered cubic (111), (200), and (220) surfaces, which is consistent with the X-ray diffraction analysis (Supplementary Fig. 6a). To clarify the activity of different crystal faces in CO oxidation, we conducted the cyclic voltammogram (CV) tests of the reaction using three single crystallographic Pt anodes (Fig. 3b), in which the Pt(111) surface gives the lowest onset potential of 0.44 V compared with that of 0.47 V on (100) and 0.50 V on (110) surfaces.

To provide insights into the activity trend of different Pt facets, density functional theory (DFT) calculations were performed to study the underlying reaction mechanism. The adsorbed CO (CO*) reacts either with OH^–^ or OH* through associative mechanisms^19,20^ (via COOH*) or with O* through a redox mechanism^12^, leading to the formation of CO_2_ (see Fig. 3c and Supplementary Methods for detailed data). Among the three reaction channels, the direct attack of CO* by OH^−^ in the solvent is most favored on all three Pt surfaces, as shown in the free energy diagrams in Fig. 3d and Supplementary Fig. 7 (see Supplementary Fig. 8 and Supplementary Table 2 for detailed data). Formation of the COOH* is the most endergonic electrochemical step and determines the overpotentials of the anode reaction, which follows the order of 0.99 V on Pt(110) > 0.68 V on Pt(100) > 0.55 V on Pt(111), in good accordance with the trend in experimental observations. Since the strength of COOH* adsorption is less dependent on the surface structure than that of CO* adsorption, as shown in Fig. 3e, the significant decrease in CO adsorption strength from Pt(110) to Pt(111) is primarily responsible for the enhanced activity on Pt(111). As the surface coordination number increases from Pt(110) to Pt(111), the *d*-band center shifts down from −2.21 to −2.44 eV, leading to reduced back-donation of the *d* electrons of Pt to the CO 2π* states (Fig. 3f). Consequently, the CO adsorption is weakened, which favors the electrochemical oxidation of CO.

### Rational design of the anode catalyst

Our theoretical calculations demonstrate that the activity of CO oxidation in EWGS process increases with the weakened interaction between CO and anode catalyst surface. Alloying Pt with other metals has been proved as an effective strategy of modulating the surface activity. Rational selection of a suitable alloying component to destabilize CO adsorption on the catalyst surface is conducted following two strategies. First, the component itself should bind CO less strongly than Pt, and thus the inert coinage metals such as Cu, Ag, and Au are chosen as potential components^21–24^, Second, the formation of alloy should shift the *d*-band center of surface Pt atoms down to a lower energy level. Considering that alloying Pt with Au and Ag will increase the lattice constant of Pt^25^ and shift the *d*-band center up^26^, Cu is finally chosen as the component to modulate the CO adsorption activity on the Pt surface. In addition, alloying with Cu will also reduce the Pt loading and hence the constituent cost for potential industrial applications. By constructing Pt_3_Cu model, we observed the decrease of lattice constant of Pt_3_Cu by 0.08 Å compared with Pt and the increase of *d*-band width of Pt_3_Cu(111) with *d*-band center shifted down to −2.47 eV, as shown in Fig. 3f. Consequently, CO adsorption on the Pt site of Pt_3_Cu(111) is weakened (Fig. 3e) and the overpotential of CO oxidation is lowered by 0.10 V compared with that of Pt(111) (Fig. 3d).

### Electrochemical performance of PtCu@CNTs catalyst

**Fig. 4:**
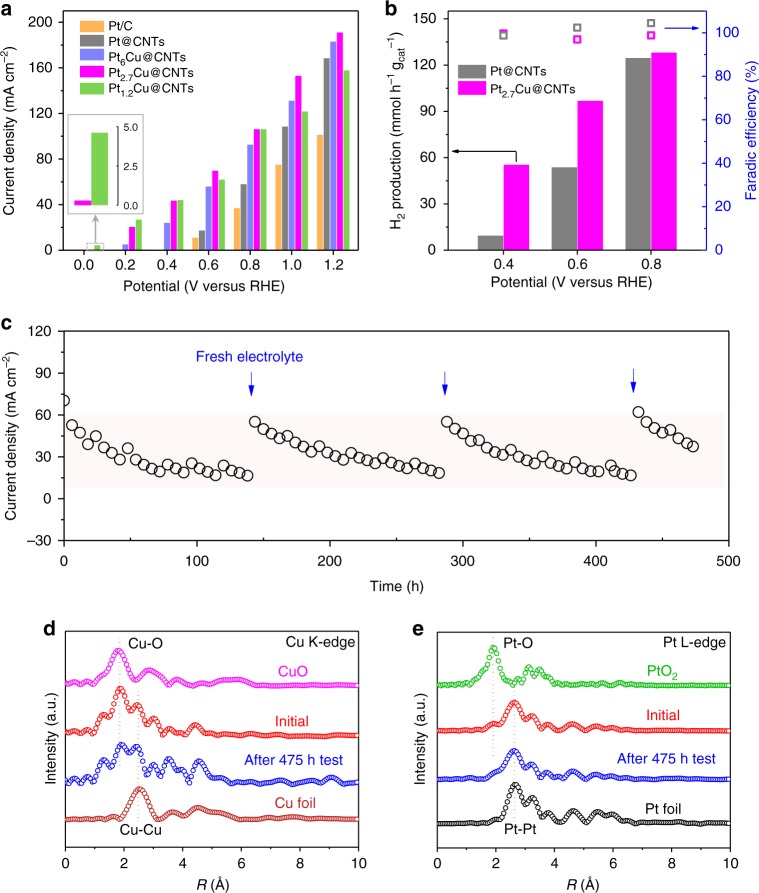
Activity and stability of the PtCu@CNTs anode catalysts for the EWGS. **a** The current density of CO oxidation on the Pt/C, Pt@CNTs, and PtCu@CNTs at different potentials. Inset shows that the current density at 0 V versus RHE reaches 0.2 and 4.6 mA cm^−2^ for Pt_2.7_Cu@CNTs and Pt_1.2_Cu@CNTs, respectively. **b** The rate of H_2_ production and faradaic efficiency on the cathode at different potentials with the Pt_2.7_Cu@CNTs and Pt@CNTs as the anode catalysts. **c** Durability test of the Pt_2.7_Cu@CNTs at a constant potential of 0.6 V versus RHE. The blue arrows denote the time of replacing the electrolyte. The tests in **a**–**c** are performed in CO-saturated 1 M KOH solution at 25 °C. **d**, **e** The *k*^2^-weighted EXAFS spectra of the Cu K-edge (**d**) and Pt L-edge (**e**) of the Pt_2.7_Cu@CNTs sample before and after 475 h of stability test, compared with those of Cu foil, CuO, Pt foil, and PtO_2_, respectively

Based on these theoretical insights, we prepared a series of PtCu@CNTs catalysts with Pt/Cu mole ratio varying from 1.2 to 6.0 (Supplementary Figs. 5, 6, and Supplementary Table 1) and measured their activity toward CO oxidation using linear sweep voltammetry. These alloy samples all exhibit lower onset potentials than that of the Pt@CNTs in the polarization curves (Fig. 4a and Supplementary Fig. 2c). The Pt_2.7_Cu@CNTs anode catalyst shows the best performance with the lowest onset potential of almost 0 V versus RHE (Fig. 4a and Supplementary Fig. 2d), leading to significant increase of H_2_ production rate on the cathode of the EWGS process compared with the case of Pt@CNTs as the anode catalyst (Fig. 4b). At a moderate operating potential of 0.6 V versus RHE, the current density of CO oxidation on Pt_2.7_Cu@CNTs reaches 70.0 mA cm^−2^, which is more than 12 times that of the pristine Pt/C catalyst without PTFE treatment (Supplementary Fig. 2c). This validates our strategies of optimizing the anode structure and catalyst in improving the anode activity. In contrast to the electrocatalytic water splitting^27–32^, the EWGS process provides a promising alternative route of hydrogen production with much lower working voltage. Moreover, the faradaic efficiencies of both electrode reactions (CO oxidation to CO_3_^2−^ and H_2_ formation) are approximately 100% in a wide range of working potential (Fig. 4b and Supplementary Fig. 9). The superior activity of the PtCu alloy catalysts is in good agreement with our theoretical predictions. X-ray absorption near edge spectroscopy (XANES) on the Pt L-edge shows a notable decrease in the white-line intensity for Pt_2.7_Cu@CNTs compared to the Pt@CNTs catalyst (Supplementary Fig. 6c), indicating a lower transition probability of electrons from the core level to the empty 5*d* bands of Pt. This is attributed to the partial filling of the 5*d* bands of Pt by the electrons transferred from Cu, which reduces the amount of empty 5*d* states and lower the *d*-band center of Pt atoms, and in turn weaken the CO adsorption on the Pt atoms as shown in the DFT calculations (Fig. 3e).

Investigation on the durability of Pt_2.7_Cu@CNTs catalyst indicated that the device can be operated stably for more than 475 h at a constant potential of 0.6 V versus RHE (Fig. 4c). During the process, formation and accumulation of carbonate ions in the electrolyte from CO oxidation will affect the reaction equilibrium and impede the reaction kinetics (Supplementary Figs. 9 and 10). Thus, the electrolyte is replaced regularly to alleviate this effect and restore the activity. XANES characterization of the Pt_2.7_Cu@CNTs catalyst after 475 h of stability test shows that the valence states of the Cu and Pt components were almost the same as that of the original catalyst, and no obvious change of the Cu–Cu bond and Pt–Pt bond in extended X-ray absorption fine structure (EXAFS) was observed (Fig. 4d, e and Supplementary Fig. 6b, c), which demonstrates the high stability of Pt_2.7_Cu@CNTs as the anode catalyst of the EWGS process.

## Discussion

In summary, we report a novel EWGS process for direct hydrogen production with over 99.99% purity and approximately 100% faradic efficiency under mild conditions. Combining experiments with theoretical calculations in the rational design of the anode structure and the catalyst composition to promote the CO oxidation reaction, the EWGS process can be driven with an anodic onset potential lowered to almost 0 V and an anodic current density of 70.0 mA cm^−2^ with long-term stability of over 475 h when operated at 0.6 V versus RHE. Considering the abundant CO feedstock, and the advantages of low-energy input and high-energy conversion efficiency in contrast to either traditional WGS reaction or the electrolysis of water, the EWGS process provides a new and promising platform to produce high purity hydrogen.

## Methods

### Preparation of catalysts

H_2_PtCl_6_·6H_2_O, CuCl_2_·2H_2_O, KOH, and CH_3_CH_2_OH were purchased from Sinopharm chemical reagent Co., Ltd. Hydroxy-functionalized multi-walled carbon nanotubes were purchased from Chengdu organic chemicals Co. Ltd. The commercial Pt/C (40 wt%) was purchased from Johnson Matthey Corp. All chemicals were used as received without further purification.

For Pt@CNTs (40 wt%) catalyst preparation, 70.0 mg of hydroxy-functionalized multi-walled carbon nanotubes were firstly dispersed into the mixture of 30 mL ethanol and 10 mL H_2_O, and then ultrasonically treated for 30 min. Subsequently, 0.239 mmol of H_2_PtCl_6_·6H_2_O solution was added drop by drop into the suspension. Afterwards, the mixture was stirred at room temperature until dry. Then the sample was transferred into a chemical vapor deposition furnace and heated with temperature programmed from room temperature to 400 °C under 50% H_2_/Ar, and was kept at 400 °C for 1 h. After cooling down to room temperature, 2% O_2_/Ar was led through to passivate the sample. The obtained catalyst was denoted as Pt@CNTs.

For preparation of the PtCu@CNTs catalysts, the H_2_PtCl_6_·6H_2_O and CuCl_2_·2H_2_O with different mole ratio were supported on the hydroxy-functionalized multi-walled carbon nanotubes by an impregnation method as used in the Pt@CNTs preparation procedure. The difference is that the PtCu@CNTs precursors were reduced under 50% H_2_/Ar at 250 °C. According to the mole ratio of Pt and Cu, the catalysts were denoted as Pt_1.2_Cu@CNTs, Pt_2.7_Cu@CNTs and Pt_6_Cu@CNTs, respectively.

### Evaluation of catalytic performance

Electrochemical measurements were performed on CHI 630, CHI 760, and PARSTAT MC (Princeton Applied Reasearch) with a three-electrode H-type full electrochemical cell equipped with a gas flow controlling system. The anode and cathode compartments were divided by a quaternary ammonium polysulfone (chloride) anion exchange membrane (aQAPS-S_6_ membrane: quaternary). The catalysts loaded on Ni foam, Pt net and Hg/HgO were used as the working electrode, counter electrode and reference electrode, respectively. The Ni foam (1 cm × 1 cm × 0.5 cm) was washed by acetone, ethanol and deionized water thoroughly before use. Firstly, 9.0 mg of catalyst was dispersed in the mixture of ethanol and deionized water with 10 µL Nafion solution (5 wt%, Du Pont) to form homogeneous ink assisted by ultrasound. Then the ink was added dropwise onto the Ni foam and dried under the infrared lamp. After that, the PTFE solution (1 wt%) was dropped on one side of Ni foam with different content as the hydrophobic layer and dried under the infrared lamp. During all the measurements, the Ni foam electrode was made as “L” type to enable part of the electrode exposed to CO/Ar atmosphere, and CO or Ar was bubbled through the electrolyte solution of the anode and cathode with a flow rate of 50 mL min^−1^. Linear sweep voltammetry with a scan rate of 10 mV s^−1^ was measured in 1 M KOH electrolyte. All the potentials have been referenced to the reversible hydrogen electrode (versus RHE). The durability test of Pt_2.7_Cu@CNTs was carried out by chronoamperometry at 0.6 V versus RHE in 1 M KOH electrolyte with CO flow rate of 50 mL min^−1^, respectively. After every 24 h, CO flow was switched to Ar for about 10 min and then back to CO again. The electrolyte is replaced every 144 h to eliminate the negative effect of the produced carbonate on the CO oxidation reaction equilibrium.

The single crystallographic Pt(111), Pt(100), and Pt(110) electrodes (with a 2 mm diameter) were annealed by CH_4_ flame and cooled in Ar and H_2_ mixture (3:1) gas atmosphere prior to each experiment. After cooling down, every electrode was quickly transferred to a clean electrochemical cell under the protection of a drop of ultrapure water. After bubbling the electrolyte with CO for a certain time, cyclic voltammetry experiments were carried out in the CO-saturated 0.01 M KOH aqueous solution on the static electrode without rotation at 25 °C.

### Gas chromatography analysis

The amount of hydrogen and carbon monoxide were quantified by the gas chromatograph (Shimadzu GC 2014) with a thermal conductivity detector (TCD) and a flame ionization detector (FID) coupled with a methanizer. Two identical MS-13X columns were connected to TCD and FID with argon and nitrogen used as the carrier gas, respectively. The faradaic efficiency (FE) of hydrogen from the cathode products was calculated by the following Eq. (4):

4 $${\mathrm{FE}}\left( {\mathrm{H}}_{2} \right) = \frac{Q_{{\mathrm{H}}_{2}}}{Q_{\mathrm{total}}} = \frac{{\mathrm{Peak}}\,{\mathrm{area}}_{({\mathrm{H}}_2)}}{\alpha } \times v \,\times t \,\times \frac{2FP_{0}}{RT} \times \frac{1}{Q_{\mathrm{total}}},$$

where *α* is a conversion factor based on calibration of the gas chromatograph with a series of standard sample, *v* is the gas flow rate, *t* is the reaction time, and *F* is the Faraday’s constant 96,485 C mol^−1^. *P*_0_ = 101.325 kPa, *R* = 8.314 J mol^−1^ K^−1^, and *T* = 298.15 K. *Q*_total_ is the total number of charges transferred in the circuit.

### DFT calculations

Spin-polarized DFT calculations were performed with the Vienna Ab-initio Simulation Package (VASP)^33^. The interaction between the ionic cores and electrons was described by the projector-augmented wave method, and the Kohn-Sham valence electronic wavefunction was expanded in a plane-wave basis set with a kinetic energy cutoff at 400 eV. The exchange-correlation effects were represented within the generalized gradient approximation using the Perdew–Burke–Ernzerhof (PBE) exchange-correlation functional^34^. The energies were converged to within 10^−^^4^ eV atom^−1^, and the forces were converged to within 0.03 eV Å^−1^.

## Supplementary information

Supplementary Information

## Data Availability

The data that support the plots within this paper and other findings of this study are available from the corresponding author upon reasonable request.
