# Supplementary material for: Room-temperature electrochemical water–gas shift reaction for high purity hydrogen production
Source: Nat Commun. 2019 Jan 8;10:86. doi: 10.1038/s41467-018-07937-w (PMC6325145; doi:10.1038/s41467-018-07937-w)
Supplement: Supplementary file 1 — Supplementary Information [file 41467_2018_7937_MOESM1_ESM.pdf]

Supplementary Information

Room-Temperature Electrochemical Water-Gas Shift Reaction for High Purity Hydrogen Production

Cui et al.

Table of Contents

Supplementary Methods..........3
Structural characterization 3
Mechanism of the EWGS reaction..........3
Computational Methods..........4
Supplementary Figures and Tables..........5
Supplementary Figure 1 | The morphology and structure of the anode. 6
Supplementary Figure 2 | Linear sweep voltammetry polarization curves for CO oxidation. ..7
Supplementary Figure 3 | Electron microscopy characterization..........8
Supplementary Figure 4 | The rate and faradaic efficiency of H_2 production..........9
Supplementary Figure 5 | Electron microscopy characterization..........10
Supplementary Figure 6 | Structure characterization. 11
Supplementary Figure 7 | Free energy diagrams for the CO oxidation reaction..........12
Supplementary Figure 8 | Optimized structures of the intermediates for EWGS reaction......13
Supplementary Figure 9 | ^{13}C NMR spectra of the anodic products. 14
Supplementary Figure 10 | The effect of different electrolyte on the CO oxidation. 15
Supplementary Table 1 | ICP analysis of the PtCu@CNTs catalysts. 16
Supplementary Table 2 | DFT calculations parameters. 17
Supplementary References 18

Supplementary Methods

Structural characterization

Scanning electron microscopy (SEM) measurements were carried out by using a Hitachi S-4800 scanning electron microscope with 15 kV accelerating voltage. High resolutions transmission electron microscopy (TEM) was conducted on FEI Tecnai F20 microscope operated at an accelerating voltage of 200 kV. The high angle annular dark field scanning transmission electron microscopy (HAADF-STEM) image and EDX mapping were carried out on the JEOL ARM200 microscope operated at 200 kV. X-ray diffraction (XRD) was performed on a Rigaku IV diffractometer with Cu Kα radiation (λ=1.5418 Å) at 40 kV and 30 mA at a scan rate of 10 ^{o} min^{-1}. X-ray absorption spectroscopy measurements were carried out at the BL14W1 beamline of the Shanghai Synchrotron Radiation Facility. Inductively coupled plasma optical emission spectroscopy (ICP-OES) was carried out on the PerkinElmer ICP-OES 7300DV. Quadrupole Mass Spectrometer EQMS-I (ShangHai Quan Tang Scientific Instruments) was used for analysis of the EWGS products from the anode and cathode. Intelligent gravimetric analyzer (IGA) test was conducted on Hiden IGA-100 instrument, and the samples were dried at 120 ^{o}C for 2 hours before test. The H_2O adsorption was tested from 0 mbar to 30 mbar at room temperature. The XANES and EXAFS were measured at the BL14W1 beamline of the Shanghai Synchrotron Radiation Facility. The slight change in the k^2-weighted spectrum of Cu after 475 hours of stability test compared to the initial catalyst may be due to the low loading of the catalyst on the electrode leading to reduced signal-to-noise ratio of the spectrum after the long time durability test.

Mechanism of the EWGS reaction

We consider the associative mechanism (I and II) and redox mechanism (III) for the EWGS reaction[1] in alkaline solution as follows:

Associative mechanism (I)

CO (g) + * $\leftrightarrow$ CO* (S1)

CO* + OH^- $\leftrightarrow$ COOH* + e^- (S2)

$COOH^* + OH^- \leftrightarrow CO_2 (g) + * + H_2O (l) + e^-$ (S3)

Associative mechanism (II)

$CO (g) + * \leftrightarrow CO^*$ (S4)

$OH^- + * \leftrightarrow OH^* + e^-$ (S5)

$CO^* + OH^* \leftrightarrow COOH^* + *$ (S6)

$COOH^* + OH^- \leftrightarrow CO_2 (g) + * + H_2O (l) + e^-$ (S7)

Redox mechanism (III)

$CO (g) + * \leftrightarrow CO^*$ (S8)

$OH^- + * \leftrightarrow OH^* + e^-$ (S9)

$OH^* + OH^- \leftrightarrow O^* + H_2O (l) + e^-$ (S10)

$CO^* + O^* \leftrightarrow CO_2 (g) + 2^*$ (S11)

Besides the COOH* intermediate, formate species (HCOO*) that has been discussed extensively as a key reactive intermediate in associative mechanism is also considered. Compared with COOH*, which interacts with water by hydrogen bonding (Supplementary Fig. 8b), HCOO* is less stable and thus less responsible for EWGS reaction on the Pt surfaces (Supplementary Fig. 8e).

Computational Methods

The Pt(110), Pt(100), Pt(111) and Pt_3Cu(111) surfaces were modeled using four-layer slabs within (2×4), (3×3), (3×3) and (4×2) surface unit cell, respectively. A vacuum region of 15 Å between any two repeated slabs was used to avoid interactions between repeated slabs along z-direction. The surface Brillouin zone was sampled with a (4 × 3 × 1), (4 × 4 × 1), (4 × 4 × 1) and (3 × 6 × 1) Monkhorst-Pack k-points grid mesh for Pt(110), Pt(100), Pt(111) and Pt_3Cu(111) surface, respectively[2]. The top two layers and the adsorbates were fully relaxed, and the remaining layers were fixed in their bulk truncated positions. The lattice constants for bulk Pt and Pt_3Cu were calculated to be 3.98 Å and 3.90 Å, in good agreement with the experimental values and previous DFT calculation[3,4].

The free energies of the intermediates of the electrochemical reactions are calculated using the computational hydrogen electrode model[5], which can be

summarized as follows:

i. By setting the reference potential to be that of the standard hydrogen electrode, we can relate the chemical potential (the free energy per H) for the reaction ($H^+ + e^-$) to that of 1/2 H_2 in the gas phase. This means that, at pH=0 in the electrolyte and 1 bar of H_2 in the gas phase at 298 K the reaction free energy of 1/2 $H_2 \rightarrow H^+ + e^-$ is zero at an electrode potential of $U = 0$.

ii. To model the water environment of the electrochemical cell, we include the effect of 3/4 (Pt(110) surface), 2/3 (Pt(100) and Pt(111) surface) and 3/8 ($Pt_3Cu(111)$) of a monolayer of water molecules on the stability of adsorbed intermediates[6,7].

iii. We include the effect of a bias on all states involving an electron in the electrode, by shifting the energy of this state by $\Delta G_U = -eU$, where U is the electrode potential.

iv. We neglect the contributions from the electric field at the electrochemical interface on the free energy of the intermediates since they have been shown to be rather small.

v. At a pH different from 0, we can correct the free energy of H^+ ions by the concentration dependence of the entropy: $\Delta G(\mathrm{pH}) = -k_B T \ln[H^+] = k_B T \ln 10 \times \mathrm{pH}$.

vi. We calculate free energies of the intermediates as $\Delta G(U, \mathrm{pH}, P_{H2} = 1\ \mathrm{bar}, T) = \Delta G_o + \Delta G_U + \Delta G(\mathrm{pH})$. ΔG_o can be expressed as $\Delta G_o = \Delta E_{DFT} + \Delta \mathrm{ZPE} + \int \mathrm{CpdT} - T\Delta S$, where E_{DFT}, ZPE, Cp and S are total energy from DFT calculations, zero-point energy, the heat capacity, and the entropy, respectively. The latter three quantities are taken from standard tables for gas-phase molecules or calculated from statistical mechanics within the harmonic approximation, using the vibrational frequencies of adsorbates and molecules as calculated within DFT (Supplementary Table 2)[8].

A correction of +0.30 eV is applied to the CO_2 molecule in the gas phase in order to account for a systematic error that originate from inaccurate descriptions of carbon-oxygen double bonds as discussed before[9]. The reversible potential of the overall half-cell reaction calculated from DFT is -0.16 V vs reversible hydrogen electrode (RHE), while agrees well with the experimental value of -0.10 V vs RHE.

$$CO + 2OH^- \rightarrow CO_2 + H_2O + 2e^-$$

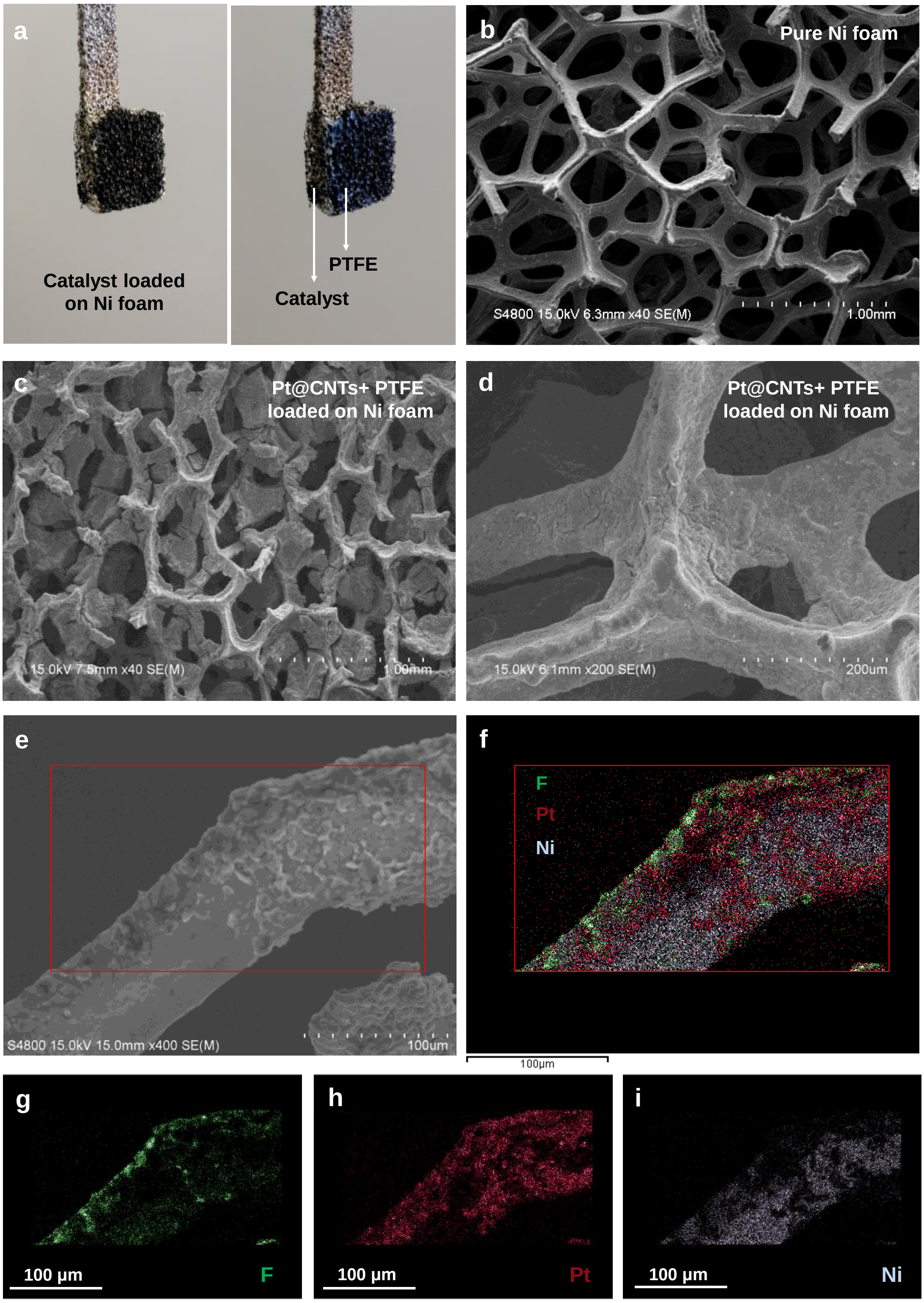

Supplementary Figure 1 | The morphology and structure of the anode. (a) Photograph of the Pt@CNTs loaded on the Ni foam with and without PTFE treatment. **(b)** SEM image of pure Ni foam. **(c-d)** SEM images of the Pt@CNTs loaded on the Ni foam after PTFE treatment. **(e-i)** SEM image of the Pt@CNTs loaded on the Ni foam after PTFE treatment, and the corresponding EDX maps for F, Pt, Ni elements, and overlapped. Scale bar: **(b-c)** 1 mm, **(d)** 200 µm, **(e-i)** 100 µm.

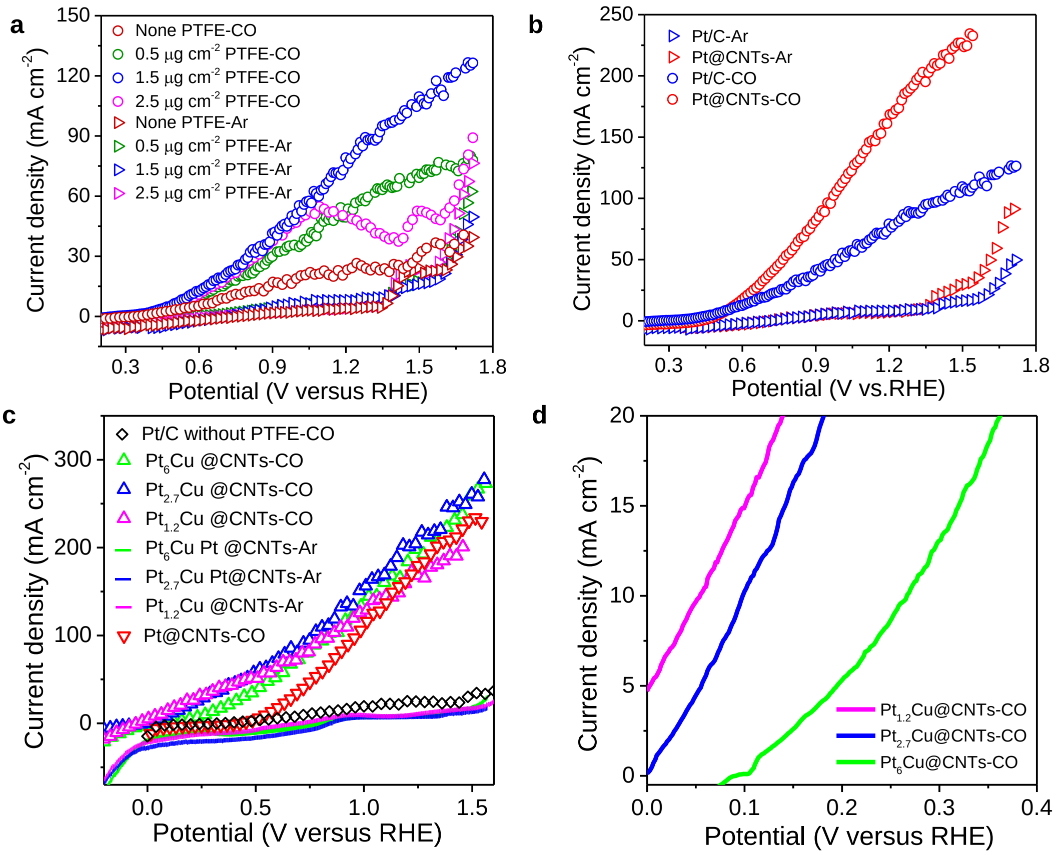

Supplementary Figure 2 | Linear sweep voltammetry polarization curves for the CO oxidation. (a) The polarization curves for the CO oxidation on the commercial Pt/C (40 wt.%) catalyst decorated with different content of PTFE in CO-saturated or Ar-saturated 1 M KOH solution at 25 °C. **(b)** The polarization curves for the CO oxidation on the commercial Pt/C and Pt@CNTs catalysts decorated with 1.5 μg cm^{-2} PTFE in CO-saturated or Ar-saturated 1 M KOH solution at 25 °C. **(c-d)** The polarization curves for the CO oxidation on the $Pt_{1.2}Cu$@CNTs, $Pt_{2.7}Cu$@CNTs, Pt_6Cu@CNTs, Pt@CNTs and Pt/C (without PTFE treatment) in CO-saturated or Ar-saturated 1 M KOH solution at 25 °C.

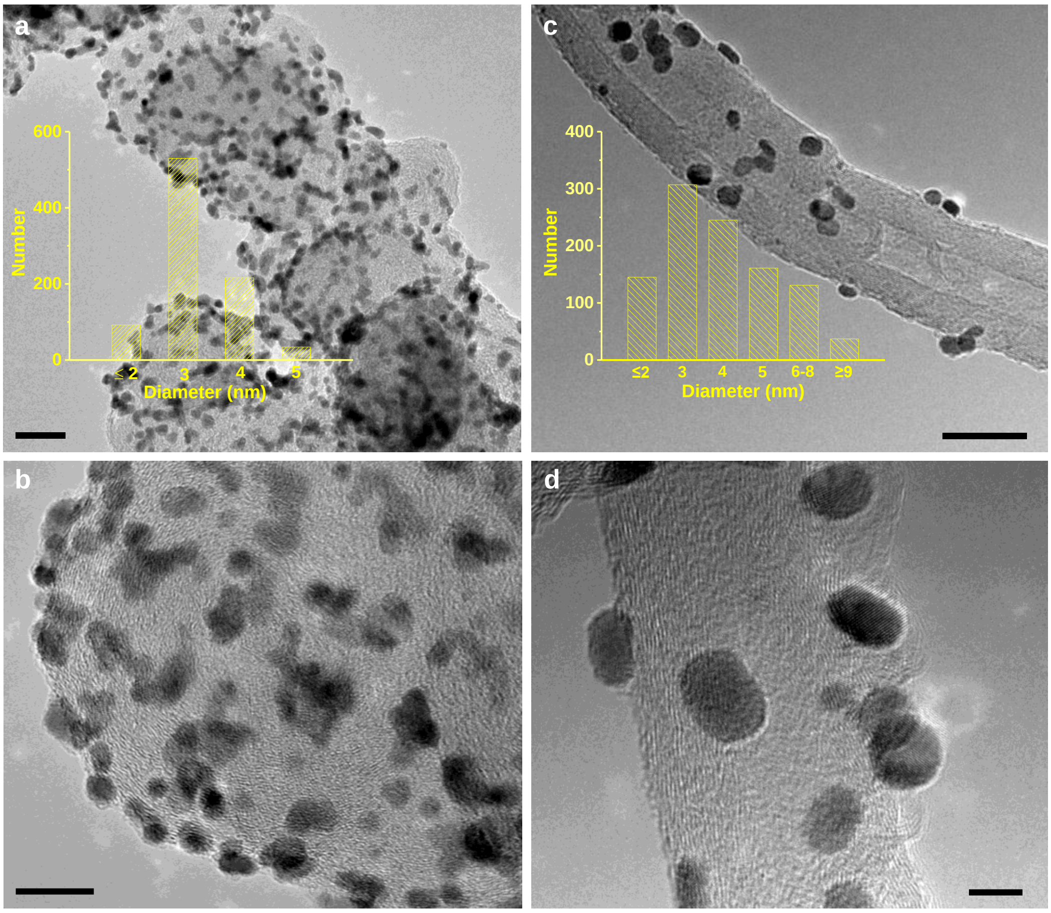

Supplementary Figure 3 | Electron microscopy characterization. (a-b) HRTEM images of the commercial Pt/C catalyst and the statistical size distribution of Pt nanoparticles. **(c-d)** HRTEM images of the Pt@CNTs catalyst and the statistical size distribution of Pt nanoparticles. Scale bar: (**a**) 20 nm, (**b**) 10 nm, (**c**) 20 nm, (**d**) 5 nm.

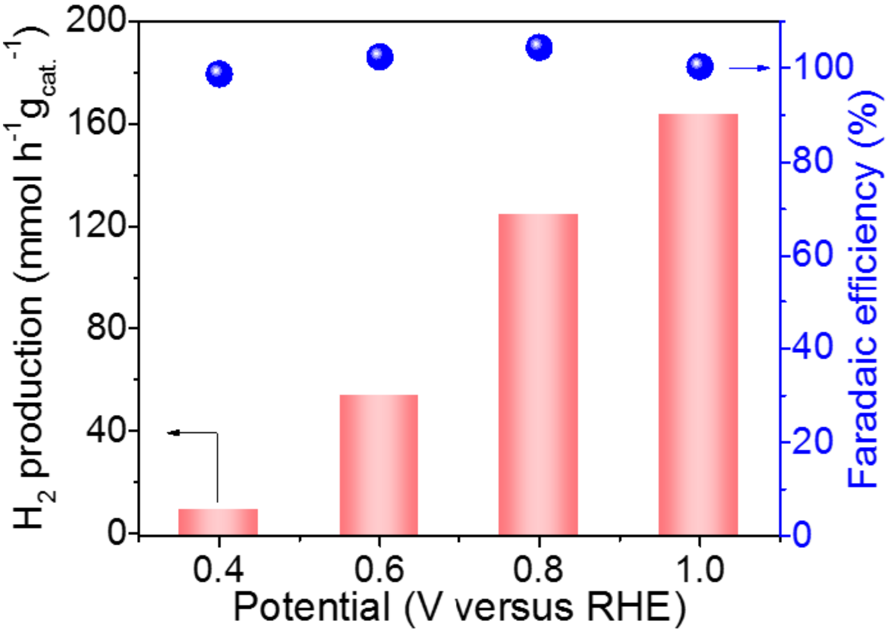

Supplementary Figure 4 | The rate and faradaic efficiency of H_2 production on the cathode at different potentials when using the Pt@CNTs as the anode catalyst. The amount of H_2 on the cathode was quantified by the gas chromatography, and the working potential varied from 0.4 V to 1.0 V versus RHE.

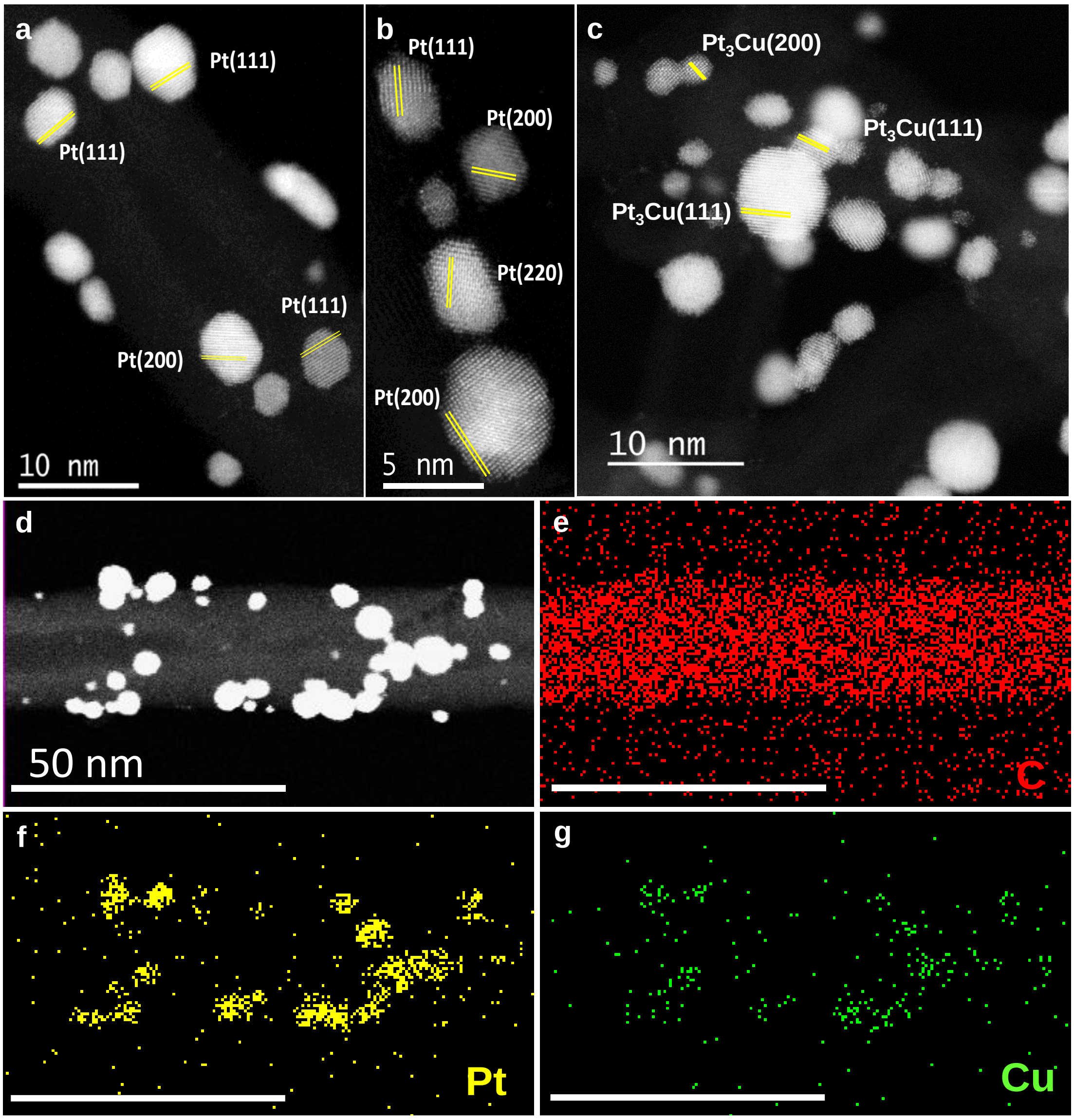

Supplementary Figure 5 | Electron microscopy characterization. (a-b) HAADF-STEM images of the Pt@CNTs sample. **(c**) HAADF-STEM image of the $Pt_{2.7}Cu$@CNTs sample. **(d-g**) HAADF-STEM image and corresponding EDX maps of the $Pt_{2.7}Cu$@CNTs sample. Scale bar: (**a**) 10 nm, (**b**) 5 nm, (**c**) 10 nm, (**d-g**) 50 nm.

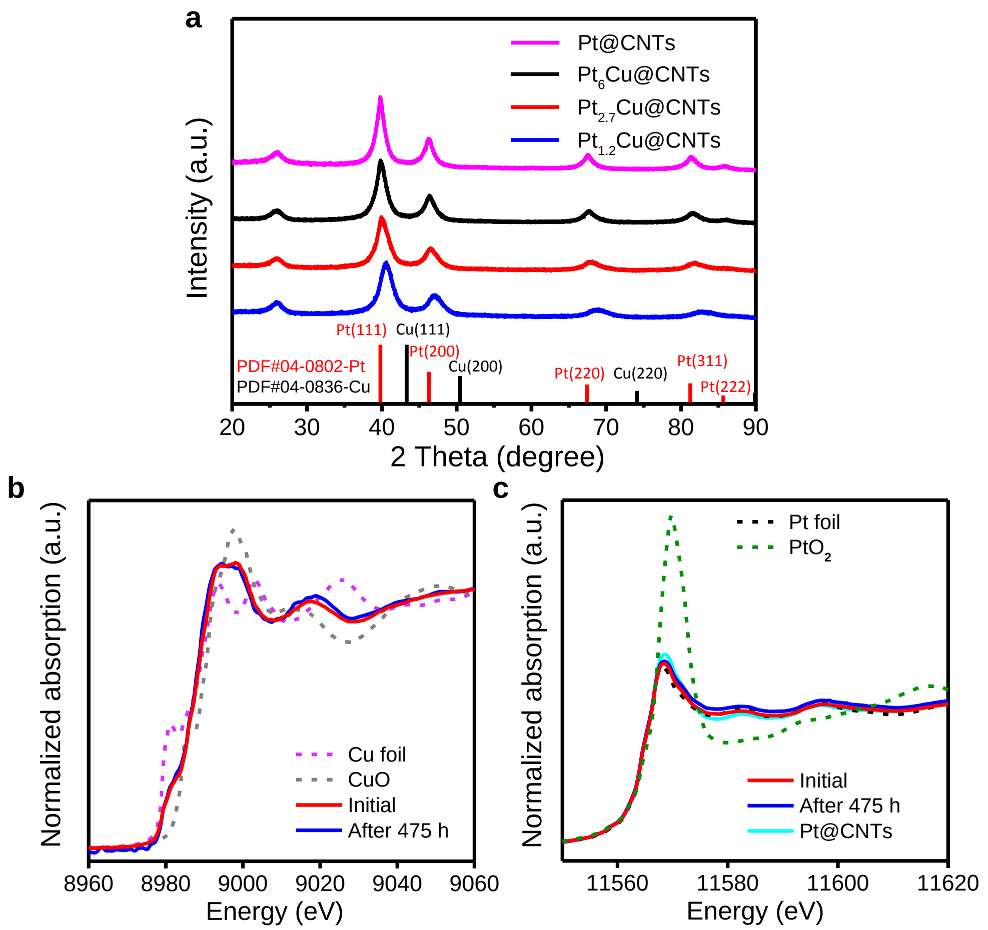

Supplementary Figure 6 | Structure characterization. (a) XRD patterns of the Pt@CNTs, $Pt_{1.2}Cu$@CNTs, $Pt_{2.7}Cu$@CNTs and Pt_6Cu@CNTs samples. **(b-c**) The normalized Cu K-edge and Pt L-edge (X-ray absorption near edge structure) of the initial and used (after 475 hours of stability test) $Pt_{2.7}Cu$@CNTs catalyst at 0.6 V compared with that of the Pt@CNTs, Cu foil, CuO, Pt foil and PtO_2.

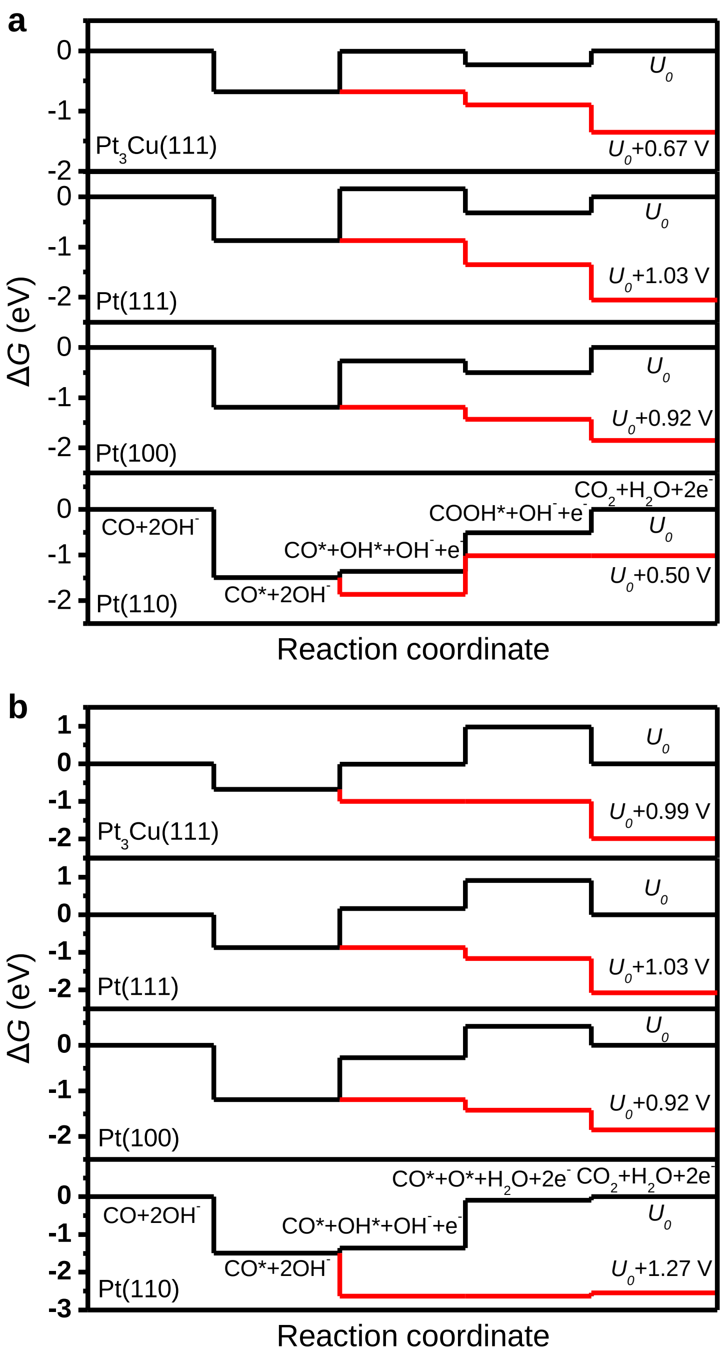

Supplementary Figure 7 | Free energy diagrams for the CO oxidation reaction. CO oxidation via Mechanism II **(a)** and III **(b)** on Pt(110), Pt(100), Pt(111) and $Pt_3Cu(111)$ at the reversible potential (U_0) of -0.16 V and at the overpotentials that all reaction steps are exothermic (except for the third step of Mechanism II and the last step of Mechanism III on Pt(110)).

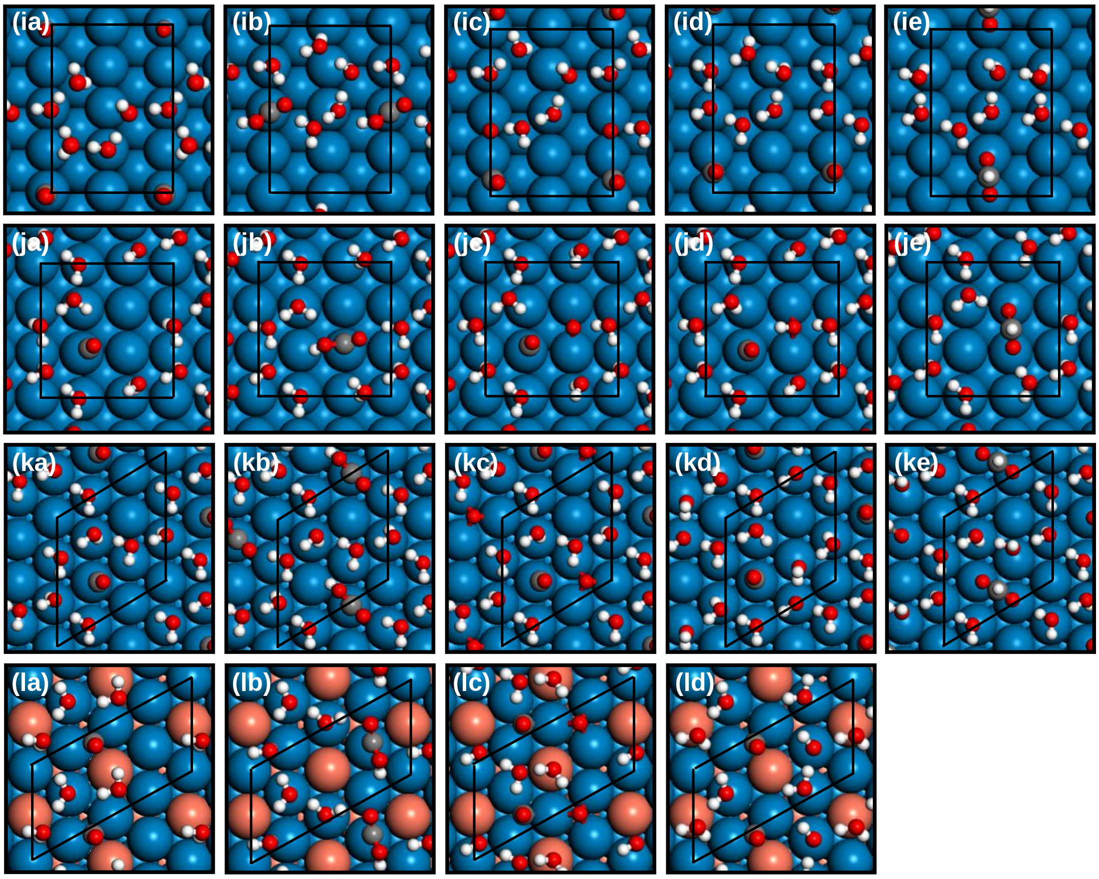

Supplementary Figure 8 | Optimized structures of the intermediates for EWGS reaction. (a) CO **(b)** COOH **(c)** CO+O **(d)** CO+OH **(e)** HCOO in the water environment on Pt(110) (i), Pt(100) (j), Pt(111) (k) and $Pt_3Cu(111)$ (l). The blue, vermilion, grey, red and white balls represent Pt, Cu, C, O and H atoms, respectively.

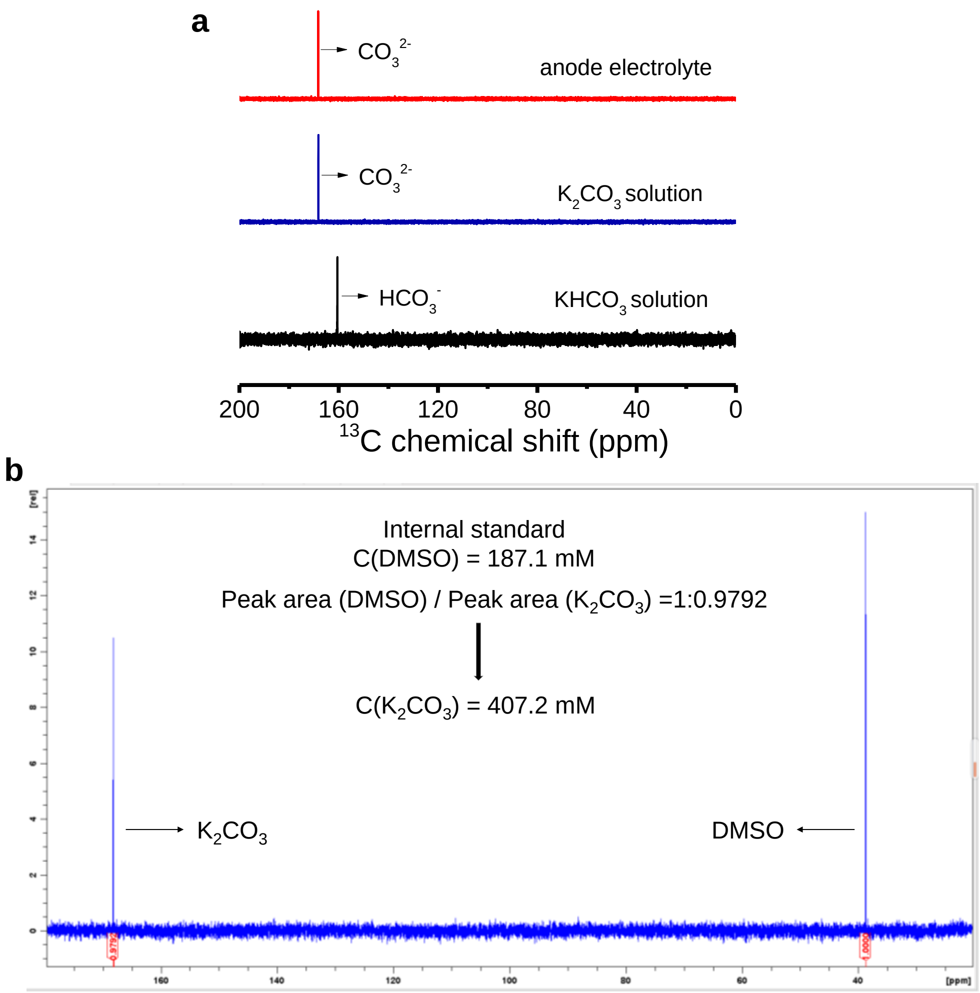

Supplementary Figure 9 | ^{13}C NMR spectra of the anodic products. (a) ^{13}C NMR spectra of the anodic electrolyte compared with K_2CO_3 solution and $KHCO_3$ solution. **(b)** Inverse-gated ^{13}C NMR spectrum of the anode compartment with the EWGS process working with a constant potential of 0.6 V versus RHE for 144 hours. The concentration of K_2CO_3 in the anode compartment is about 407 mM calculated by the internal standard dimethyl sulfoxide (DMSO), far below the concentration of saturated potassium carbonate solution at 25 ^{o}C, i.e. 8.3 M. The inverse-gated ^{13}C NMR spectra were acquired with a recycle delay of 50 s, and the test procedure was optimized by a standard mixture of K_2CO_3 (176.9 mM) and DMSO (182.3 mM).

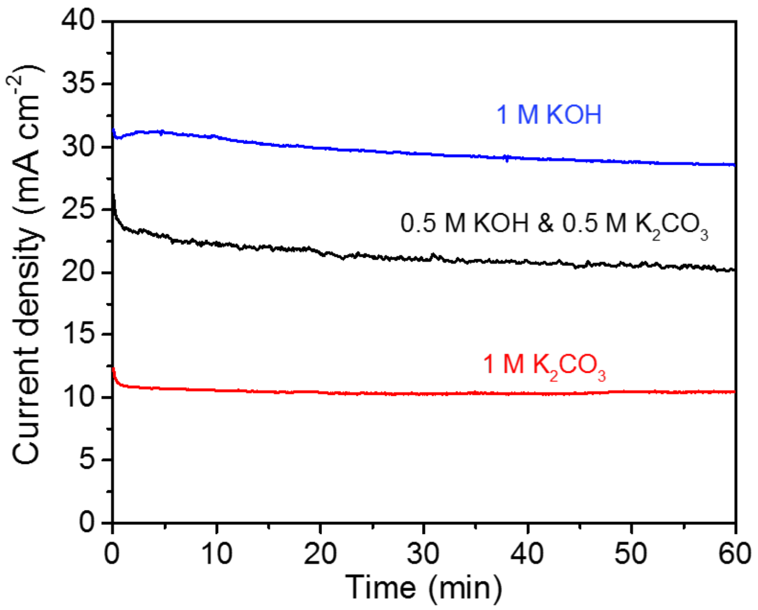

Supplementary Figure 10 | The effect of different electrolyte on the CO oxidation in EWGS process. Chronoamperometric experiments for the Pt@CNTs at a constant potential of 0.6 V vs. RHE in CO-saturated 1 M KOH, 1 M K_2CO_3, the mixture of 0.5 M KOH and 0.5 M K_2CO_3 solution at 25 ^{o}C, respectively.

Supplementary Table 1 | ICP analysis of the PtCu@CNTs catalysts.

Sample	molar ratio n(Pt)/n(Cu)
Pt_6Cu@CNTs	5.96
$Pt_{2.7}Cu$@CNTs	2.68
$Pt_{1.2}Cu$@CNTs	1.23

Supplementary Table 2 | Zero-point energies (ZPE), enthalpic temperature corrections (∫CpdT) and entropy corrections (TS) for the reaction species at 298 K. The entropies of H_2(g), CO (g) and CO_2 (g) are calculated at 1 atm, while the entropy of H_2O (g=l) is calculated at 0.035 atm, which corresponds to the vapor pressure of liquid water.

Species	Pt (110)			Pt (100)			Pt (111)			Pt_3Cu (111)		
	ZPE	∫CpdT	TS	ZPE	∫CpdT	TS	ZPE	∫CpdT	TS	ZPE	∫CpdT	TS
H_2(g)	0.27	0.09	0.41	0.27	0.09	0.41	0.27	0.09	0.41	0.27	0.09	0.41
H_2O(g)	0.57	0.10	0.67	0.57	0.10	0.67	0.57	0.10	0.67	0.57	0.10	0.67
CO(g)	0.13	0.09	0.61	0.13	0.09	0.61	0.13	0.09	0.61	0.13	0.09	0.61
CO_2(g)	0.31	0.12	0.66	0.31	0.12	0.66	0.31	0.12	0.66	0.31	0.12	0.66
CO*	0.21	0.07	0.16	0.21	0.07	0.14	0.21	0.06	0.13	0.20	0.06	0.11
CO*+O*	0.28	0.09	0.16	0.28	0.10	0.18	0.29	0.09	0.16	0.26	0.09	0.15
CO*+OH*	0.58	0.10	0.19	0.59	0.11	0.20	0.60	0.10	0.18	0.57	0.10	0.18
COOH*	0.63	0.09	0.16	0.61	0.10	0.20	0.62	0.10	0.20	0.62	0.10	0.19

Supplementary References

1. Hansen, H. A., Varley, J. B., Peterson, A. A. & Nørskov, J. K. Understanding trends in the electrocatalytic activity of metals and enzymes for CO_2 reduction to CO. *J. Phys. Chem. Lett.* **4**, 388-392 (2013).
2. Monkhorst, H. J. & Pack, J. D. Special points for Brillouin-zone integrations. *Phys. Rev. B* **13**, 5188-5192 (1976).
3. Ma, Y. & Balbuena, P. B. Pt surface segregation in bimetallic Pt_3M 1 alloys: A density functional theory study. *Surf. Sci.* **602**, 107-113 (2008).
4. Wyckoff, R. W. G. *Crystal Structures*, 2nd Edition (Wiley: New York, 1963).
5. Nørskov, J. K. *et al.* Origin of the overpotential for oxygen reduction at a fuel-cell cathode. *J. Phys. Chem. B* **108**, 17886-17892 (2004).
6. Ogasawara, H. *et al.* Structure and bonding of water on Pt(111). *Phys. Rev. Lett.* **89**, 276102 (2002).
7. Carrasco, J. *et al.* A one-dimensional ice structure built from pentagons. *Nat. Mater.* **8**, 427-431 (2009).
8. Zeng, Z. H. *et al.* Towards first principles-based prediction of highly accurate electrochemical pourbaix diagrams. *J. Phys. Chem. C* **119**, 18177-18187 (2015).
9. Studt, F. *et al.* The mechanism of CO and CO_2 hydrogenation to methanol over Cu-based catalysts. *ChemCatChem* **7**, 1105-1111 (2015).
